# Health-BlockEdge: Blockchain-Edge Framework for Reliable Low-Latency Digital Healthcare Applications

**DOI:** 10.3390/s21072502

**Published:** 2021-04-03

**Authors:** Muneeb Ejaz, Tanesh Kumar, Ivana Kovacevic, Mika Ylianttila, Erkki Harjula

**Affiliations:** Erkki Koiso-Kanttilan Katu 3, University of Oulu, Linnanmaa, 90570 Oulu, Finland; Tanesh.Kumar@oulu.fi (T.K.); ivana.kovacevic@oulu.fi (I.K.); mika.ylianttila@oulu.fi (M.Y.); erkki.harjula@oulu.fi (E.H.)

**Keywords:** IoT, healthcare, remote monitoring, edge computing, blockchain, fog computing, mist computing, energy efficiency

## Abstract

The rapid evolution of technology allows the healthcare sector to adopt intelligent, context-aware, secure, and ubiquitous healthcare services. Together with the global trend of an aging population, it has become highly important to propose value-creating, yet cost-efficient digital solutions for healthcare systems. These solutions should provide effective means of healthcare services in both the hospital and home care scenarios. In this paper, we focused on the latter case, where the goal was to provide easy-to-use, reliable, and secure remote monitoring and aid for elderly persons at their home. We proposed a framework to integrate the capabilities of edge computing and blockchain technology to address some of the key requirements of smart remote healthcare systems, such as long operating times, low cost, resilience to network problems, security, and trust in highly dynamic network conditions. In order to assess the feasibility of our approach, we evaluated the performance of our framework in terms of latency, power consumption, network utilization, and computational load, compared to a scenario where no blockchain was used.

## 1. Introduction

The emergence of ubiquitous and pervasive computing and recent advancements in wearable and smart sensing technologies is revolutionizing the conventional modes of accessing and delivering healthcare services [1]. In addition to that, the advent of 5G and relevant enabling technologies provides several opportunities for the rapid developments of future healthcare systems [2,3]. For example, the traditional means of measuring patients’ health parameters and vital signs are being replaced by automatic medical sensing technologies (medical sensors) [4]. Furthermore, technology development has facilitated healthcare-related processes, such as patient registration to the hospital, keeping track of their electronic medical/health records (EMR/EHR), and authorized access to these records.

Future healthcare systems require a secure, trusted, and dynamic service and computing environment, i.e., “personalized and connected health” [5]. These smart and connected healthcare systems are expected to provide advanced medical services, such as advanced diagnostic, remote and real-time patient monitoring, efficient handling mechanisms for the healthcare big data, and digital solutions for addressing sudden challenges such as global pandemics [6,7]. Thus, the design of future digital healthcare systems must fulfill the resulting high requirements, for instance in terms of providing secure and trusted mechanisms for healthcare data sharing, data management of the massive data, and ensuring ubiquitous availability of the needed services in the desired amount of time. To fulfill these requirements, recent network and communications-related enabling technologies are going to play a significant role [8].

With the change in the demographics of the world population, the aging of people is becoming a key challenge for future healthcare services [9]. The expenses of providing the needed digital healthcare infrastructure for the elderly population are expected to rise in the future. Most elderly people want to live independently as long as possible. Since many of them live far from hospitals, one of the crucial requirements is to provide safe, secure, and timely remote healthcare monitoring services [10,11]. In addition, future hospitals are likely to have the additional burden of patients with chronic diseases who require continuous care for longer periods. Furthermore, in the case of highly contiguous diseases, (such as the recent COVID-19 pandemic) [12], it is desirable to handle patients with mild symptoms remotely at their home.

Edge computing is considered as a vital technology enabler in providing time- dependent or delay-critical healthcare services, specifically in the case of emergencies, real-time patient monitoring (intensive care unit (ICU) patients), and for faster data analysis of patients with conditions requiring fast medical response or contiguous diseases [13,14]. This brings computational and processing resources near to the end-users and devices to perform necessary real-time data analysis and decision-making functions and to improve resource-efficiency by reducing the amount of data transferred between the end systems and centralized cloud servers [15,16].

The blockchain is yet another promising technology in the context of the future healthcare domain and can provide a number of key characteristics in terms of decentralization, traceability, transparency, and immutability [17]. In addition, it enables a trusted computing environment required for the involved network entities/healthcare stakeholders to securely share information and resources among them [18]. For example, EMR/EHR records can be securely managed and monitored by the blockchain, and only authorized stakeholders are given access to append or retrieve the data. Some of the potential blockchain applications in the healthcare sector include: clinical data sharing, maintaining medical history, drug supply chain management, and billing/insurance claims, among others [19,20]. Therefore, in this paper, we integrated blockchain with a three-tiered IoT edge architecture for a elderly remote monitoring use case to ensure trusted data sharing among different healthcare stakeholders, tracking or monitoring of various processes and their phases, and maintaining the medical history of senior citizens, among others.

In this direction, this paper focused on the integration of the blockchain and edge computing and their combined impact on the efficacy and efficiency of remote health monitoring systems, which furthermore contributes to the overall efficacy and efficiency of the digital healthcare system. The core aim behind combining these two enabling technologies is to fulfil the crucial requirements of remote monitoring use cases, including, e.g., delay-critical monitoring of patients’ vital signs, requiring low-latency, trusted, and privacy-preserving automated data management and decision-making. The main contributions of this paper include:

- A blockchain-edge-based conceptual network framework for remote healthcare monitoring applications;
- The performance and efficiency evaluation of the proposed framework and comparison to a baseline architecture without the blockchain; and
- The analysis of the achieved results and their real-world impact.

The rest of the paper is organized as follows: Section 2 presents the existing work related to the blockchain and edge computing for healthcare applications. Section 3 introduces the proposed Health-BlockEdge framework and the central performance and efficiency parameters in the context of a remote patient monitoring use case. In Section 4, the proposed framework is evaluated against a baseline setup without blockchain. Section 5 provides a discussion and future research directions, and finally, Section 6 concludes the paper.

## 2. Related Work

### 2.1. Edge and Fog Computing

Edge computing brings computational and processing resources near to the end-users and devices to perform necessary real-time data analysis and decision-making functions and to improve resource efficiency by reducing the amount of data transferred between the end systems and centralized cloud servers [15,21]. In addition, the inclusion of the edge network improves the data privacy, security, and reliability of the systems running on the edge architecture [22]. Multi-access Edge Computing (MEC) is the European Telecommunications Standards Institute’s (ETSI) standard for unleashing the full potential of low-latency 5G radio communications in the mobile access network [23]. MEC brings cloud computing 1–2 hops away from the IoT devices, which include the cellular base station or access point [21].

Fog computing [24] is a term closely related to edge computing, the distinction between these two terms being vague due to various overlapping definitions in the literature. We define the difference as follows: EC refers to the computational edge infrastructure and FC to the distributed service architecture above the edge computing infrastructure and local nodes. FC typically covers functions such as caching, data processing, and analytics occurring near the source of the data that improve the performance at the edges of the network, reduce the burden on data centers and core networks, and improve the resilience against networking problems [24,25,26].

### 2.2. Local Edge and Mist Computing

In mission-critical applications, the role of the local network in delivering the most critical services is emphasized in the cases when the connection to the global network is not available or not stable [25]. Mist computing refers to the distributed service architecture above the local edge computing infrastructure and local nodes [27].

Complex sensors like surveillance cameras and healthcare monitoring devices are composed of a micro-controller or microcomputer that can perform certain tasks locally [28]. Although these devices have limited processing power, this network layer has not yet been explored by scientists and researchers. With more advanced health monitoring and imaging solutions, such as MRI scanners, the available computational capacity is much higher compared to traditional IoT devices, allowing also more demanding edge computing tasks to be deployed at the local level. The only disadvantage involved in mist computing lies in its complexity. The devices used for mist computing are usually application specific, and the sensors are often heterogeneous, making the implementation of a solution more complicated.

The authors in [29] proposed an efficient and secure mist computing framework that ensures the requirements of a recently released public geospatial heath data set. The aim of the model is to enhance the security features with the help of mist nodes for effective management of geospatial health data. The proposed prototype was evaluated and the results compared with the traditional cloud framework. Furthermore, in [30], a mist-fog computing framework for the Internet of Healthcare Things (IoHT) was proposed. The model achieved ultra-low latency with the assistance of mist-fog computing in the healthcare monitoring system.

### 2.3. Blockchain

In the early days of the blockchain, it was originally used mainly as a technology for banking and finance applications, e.g., cryptocurrency [31]. Bitcoin was the first cryptocurrency, implemented by Satoshi Nakamoto in 2009 [32]. Since then, the blockchain has been a significant technology enabler for various IoT applications due to the number of key characteristics it provides, e.g., decentralization, immutability, transparency, and trusted computing environments [33]. Some of the well-known blockchain-based IoT applications include: smart healthcare, transportation, supply chain management, and the Industrial IoT (IIoT) [34]. The blockchain belongs to distributed ledger technologies (DLT), where all transactions are replicated and recorded among all involved parties in a peer-to-peer network architecture and are secured using a strong cryptographic mechanism [35].

The blockchain has significant utility in various areas of the healthcare domain, e.g., secure data sharing among various healthcare entities, maintenance of healthcare records, remote monitoring of patients, pharmaceutical supply chain management, and health insurance claims, among many other areas [19,36,37]. The blockchain enables various key features in healthcare, such as a fine-grained access control mechanism for secure access to healthcare records, the needed distributed trust among various healthcare entities (patients, healthcare professionals, hospital administration, and service providers), authentication, traceability, process tracking and monitoring, and data privacy protection [38,39,40]. In addition, the blockchain offers valuable opportunities in terms of trusted data management mechanisms for digital healthcare systems [41,42,43,44]. However, despite its many desirable features from the viewpoint of healthcare applications, there are still concerns related to, e.g., the achievable performance and efficiency such as ensuring low latency, enhanced scalability, and the increased storage capabilities (ledger size) [45,46]. In this paper, we explored addressing these challenges by integrating it with edge computing to achieve higher performance and efficiency.

## 3. The Evolution of Cloud IoT Models

Cloud computing is evolving from a fully centralized computational model towards more decentralized edge-cloud models [47]. In the following subsections, we briefly introduce the three fundamental architectural cloud IoT models.

### 3.1. Traditional Cloud IoT Model

**Figure 1:**
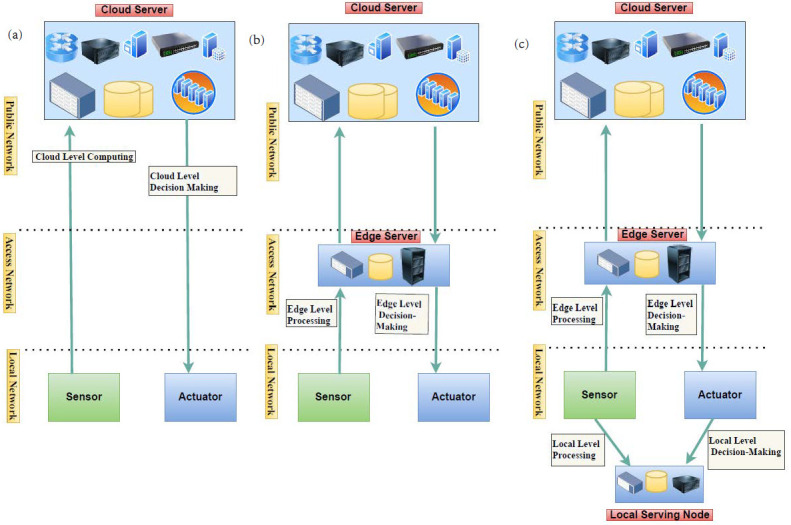
Existing IoT models: (**a**) cloud IoT model; (**b**) edge-cloud IoT model; (**c**) local edge-cloud IoT model.

Figure 1a highlights the traditional cloud-based IoT architecture, which consists of three main tiers: core, access, and local tier [47]. The lowest tier of the cloud IoT model is the local tier, which involves low-power end-devices such as sensors and actuators (e.g., instrumentation and monitoring devices) that sense the data and interact with the surroundings. The middle tier is the access tier, which is comprised of different gateway devices (access points, switches, routers, etc.) connecting local devices to core networks. The uppermost tier is the core tier, which includes the servers at the data centers, as well as high-performance switches and routers to deliver the data to/from lower tiers. Cloud servers handle all application logic, decision-making, data-management, and storage functions in this model.

### 3.2. Edge-Cloud IoT Model

Figure 1b illustrates the edge-cloud IoT model, where the access tier is—in addition to its role connecting the local tier to the core tier—considered as a middle tier in the cloud IoT system, handling a part of the cloud services closer to the end-nodes (i.e., IoT devices). It reduces the physical distance between IoT devices and the computation processing server and, therefore, also provides lower latency between the end-device and the processing unit, compared to centralized cloud-based computation. It also reduces the processing burden on centralized cloud servers by performing some of the data processing at the access layer and therefore closer to the end-users. This model is also more resilient to core network problems and therefore more reliable to fulfill the requirements of mission-critical applications. Furthermore, security is improved by limiting the data propagation within the access network when needed. Together, these new features enable real-time and mission-critical cloud applications and services.

### 3.3. Local Edge-Cloud IoT Model

In our previous work, we proposed a local edge-cloud IoT model [25,47,48], which utilized the capacity of local nodes at the local tier to manage some parts of the processing and data analytics as presented in Figure 1c. This model allows a part of the computation and decision-making to take place in local nodes with sufficient computational capacity and stability. Therefore, in this model, the cloud applications and services and their parts can be deployed to the most suitable of three network tiers: local, access, and cloud. To enable this scenario, we introduced the concept of nanoservices [25,49]. A nanoservice is a lightweight microservice, which, with resource-aware orchestration, can be deployed on resource-constrained IoT nodes. We aimed at developing the nanoservice concept towards full compatibility with current cloud microservice systems. The local edge-cloud IoT model improves security and privacy by allowing the analysis of sensitive data to be managed locally instead of sending them to public servers for analysis. Furthermore, the model enables deploying most critical functions locally, therefore improving their resilience to access network problems and context awareness through available local sensor data.

The authors in [50,51,52] introduced a two-tier IoT model using the iFogSim simulator and analyzed the network parameters (including latency, power consumption, network usage, cost). Furthermore, in one of our previous works [47], we exploited the benefit of local tier processing, proposed a three-tier IoT edge model, and analyzed the performance of the network. In [53], we extended the work performed in [47] by integrating the blockchain technology in the three-tier IoT edge models and evaluated the performance with and without the addition of the blockchain. However, in this paper, we extended our previous research [53] for the delay-critical healthcare use case and measured a number of key parameters such as network latency, power consumption, network usage, total cost, and number of operations executed in order to evaluate the overall performance of our proposed framework.

## 4. Health-BlockEdge Concept

This paper extends the work in [53,54] by proposing the conceptual BlockEdge framework for the remote healthcare monitoring use case. This conceptual framework is called Health-BlockEdge. We provide an overview of the concept and analyze its performance in a healthcare-related scenario by comparing two scenarios: one with the blockchain and the other without the blockchain. In the following subsections, we present the use case and the conceptual framework in detail.

### 4.1. Use Case: Remote Healthcare Monitoring

In order to analyze the proposed Health-BlockEdge concept, we used a remote healthcare monitoring use case. In the use case, the activity and various health parameters of an elderly person living at home were monitored remotely. Another remote care example could be the case of a contagious disease, such as COVID-19, where remote home care effectively prevents the disease from spreading through the physical contact of the patient with others. In this case, the patients with mild symptoms can be treated and monitored remotely from home.

The monitoring includes the tracking of the patient health parameters, activity, and behavior through smart sensors and devices. In the case of exceptional situations, such as a detected accident or a health parameter (such as blood pressure, oxygen level, blood sugar, etc.) going outside the normal range, the system can notify a healthcare professional, who can further analyze the health conditions and accordingly provide recommendation of advanced treatment or hospitalization if necessary.

### 4.2. Model Overview

The framework consists of three tiers, i.e., local tier, access tier, and core tier.

**Figure 2:**
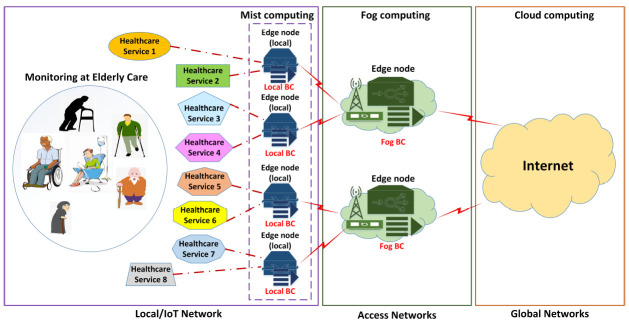
Blockchain-Edge framework for remote elderly healthcare monitoring.

Local tier: The local tier is comprised of numerous sensors and devices, including on-body/in-body medical sensors/devices that can measure health-related data/parameters (vital signs), do the basic data-prepossessing and analysis, and forward this to the assigned high-computational nodes/servers (edge and cloud servers). Figure 2 depicts multiple healthcare services that need to be delivered to remote patients. The nature of a particular healthcare service depends on the need/requirement of the users/patients in elderly care. For example, “Healthcare Service 1” may contain medical sensors that provide services related to measuring the heart rate. Likewise, “Healthcare Service 2” can be related to the video surveillance/monitoring of an elderly person.

The resource-constrained medical nodes at the local tier are connected to the higher capacity computational nodes (local edge nodes) for local data processing, analysis, decision-making, and forwarding the information/data further to the edge or the global networks. The local edge nodes are located in the same location, i.e., at the home of a disabled person or at the elderly care home. In addition, another major task of these high-resource-capable local edge nodes is to provide the needed resources and computational capabilities to run the local blockchain. A consortium/permissioned blockchain will run at the local network (at the local edges) to ensure trusted healthcare data/information sharing among various entities (users, doctors/staff, service providers, emergency units) in the network. The local network can be seen as the connection between the resource-constrained IoT medical sensors and devices and the local edge nodes that can perform the required sensing, collection, local data pre-processing, and decision-making and send the request of the high-computational tasks to the higher tiers. Access tier: In comparison with the local tier, the access tier is considered much richer in terms of the resources/computational capabilities. The access tier includes edge servers, i.e., MEC servers, that provide computational resources for remote monitoring services, as shown in Figure 2. This tier enables the crucial and demanding computational features such as AI-based data analytics and decision-making, adaptive/customized security and privacy solutions, dynamic allocation/orchestration of the available resources, etc. At this tier, a public/permissionless blockchain is run to share the necessary patient information or keep a record for resource sharing among various edge nodes. The structure of the preceding sentence is a bit unclear: the blockchain can also provide the resources for the auction/renting functionality in various entities in the network and can trade various resources, i.e., resources may include computational/processing capabilities, storage capabilities, or using of hospital resources (e.g., ambulances and workforce).

Core tier: The core tier includes the global Internet core architecture and the cloud data centers providing a practically infinite amount of resources and computational capacity for cloud services. The key role of the global tier is to provide the highest layer service logic to manage and supervise the overall phases/processes of the healthcare systems and can provide the needed resources. In the case of the traditional cloud IoT model, all services, data management functions, etc., are managed at the data centers.

## 5. Performance Metrics

In this section, we present the key performance factors used to evaluate the efficiency of the proposed scheme.

Latency: We define latency in the IoT computational offloading as the time between the moment an observed event has occurred and the moment of the system’s response to this event. Total latency *L* is the sum of the communication and computational latency, so we can write:

(1) $$L=L_{U}+L_{C}+L_{D},$$

where $L_{U}$ is the time to upload the computational task/data to the cloud/fog/local device for processing/storage, $L_{C}$ is the computational latency for task execution, and $L_{D}$ is the communication delay of the control message/result of the computation from the server to the IoT node.

Power consumption: This refers to the power consumption of data forwarding, computation, and data storage at each network layer. The power consumption *P* of the task execution can be expressed as:

(2) $$P=P_{C}+P_{E}+P_{L}+P_{N}.$$

Here, $P_{C}$ is the power consumption of the computational infrastructure (servers) at the core level, $P_{E}$ the power consumption at the edge level, $P_{L}$ the power consumption at the local level, and $P_{N}$ the power consumption of the communication infrastructure of the network.

Network usage: The network usage can be referred as the utilization of each of the three network layers in the defined healthcare use case. It is measured as the number of MB/s transmitted over the communication networks. The network usage increases with the increase of the number of data processing and network devices.

Total cost: The total cost of the system *C* is the sum of the communication (network) cost $C_{net}$ and the computational (server) cost $C_{comp}$.

(3) $$C_{T}=C_{net}+C_{comp}$$

Here, $C_{T}$ is the total cost, $C_{net}$ the communication cost, and $C_{comp}$ the computation cost.

Communication cost: This depends on the amount of packets relayed through the network and the cost per packet:

(4) $$C_{net}=\sum_{n=1}^{N}*\frac{D_{n}}{l}*C_{p}*\left(I_{n}-1\right)$$

Here, *N* is the number of devices, $D_{n}$ the size of the sensed data (MB) at device *n*, *l* the size of the packet (MB/packet) defined by the provider, $C_{p}$ the cost of forwarding each packet (x/packet), and *I* the number of nodes on the path between the IoT sensor *n* and the server processing the data from sensor *n*. Here, x is a value that represents the cost.

Computational cost: This refers to the cost of the resources (CPU, power, storage, memory) used in each computational node in the network.

(5) $$C_{comp}=\sum_{k=1}^{K}\left[CM*MUk+C_{S}*ST_{k}+C_{P}*P_{k}+C_{mips}*S_{k}\right]$$

Here, *K* is the number of devices (cloud/edge/local used for computation) in the network, $C_{M}$ the cost per memory (x/GB), $MU_{k}$ the memory used (GB), $C_{S}$ the cost per storage (x/MB), $ST_{k}$ the storage consumed (MB), $C_{P}$ the cost per power (x/W), $P_{k}$ the power consumption (W) of server *k*, $C_{mips}$ the cost per MIPS(x/mips) allocated, and $S_{k}$ the total size of the algorithm executed at *k* in millions of instructions (MI).

Total number of operations executed: This is the sum of all operations necessary to execute the sensed data processing algorithm. In order to execute the algorithm, the system needs to orchestrate resources and to process the task at the server, as well as perform control operations in the communication network for sending the sensed data from the end node IoT sensor to the server.

(6) $$Op=\sum_{n=1}^{N}\sum_{i=1}^{I_{n}}\left(H_{i}\right)+R_{n}+S_{n}$$

Here, $H_{i}$ is number of control plane operations for handling the task $S_{n}$ (receiving, pre-processing, forwarding operations) by each device on the path between end-devices (such as gateways, WiFi, routers/switches, etc.) and $R_{n}$ the number of operations executed to orchestrate the resources of the server for the execution of the algorithm with $S_{n}$ number of instructions.

## 6. Results

We evaluated the performance and efficiency of our proposed framework on the three IoT models and compared the results with the case where the blockchain was not in use [25]. We considered the following performance key factors as the evaluation metrics: (1) latency; (2) power consumption; (3) network usage; (4) total cost; and (5) total number of operations executed required to realize the full potential of the traditional cloud, edge IoT, and fog for real analytics. These performance metrics were described in more detail in the previous section.

### 6.1. Evaluation Setup

There are a number of simulation tools available, but only a few tools are capable of analyzing the performance of the fog and edge computing scenarios. We used the iFogSim simulator for our evaluations. It provides a high-level hierarchy, and the key reason for choosing this tool is that it provides application placement policies at different layers in the network and allows simulating real-time applications. The simulations were carried out by using a remote patient health monitoring use case and comparing the results of the three IoT models, with and without the blockchain.

**Figure 3:**
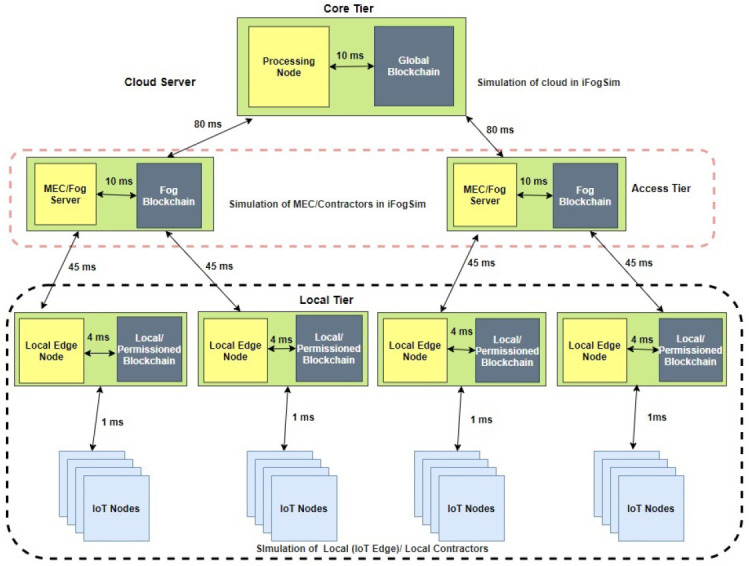
High-level design of the Health-BlockEdge framework in iFogSim. MEC, Multi-access Edge Computing.

Figure 3 illustrates the Health-BlockEdge architecture implemented in the iFogSim simulation. We analyzed three different scenarios. The algorithm that continuously analyzes the sensed patient’s data can be placed at the local tier, access tier, or cloud tier. We implemented these scenarios with the blockchain and compared the results with the system without the blockchain.

We modeled the local tier of the system in iFogSim by deploying N=4 resource-constrained IoT nodes and four local edge nodes, together with the lightweight blockchain. The local/permissioned blockchain allowed the data to be exchanged with other edge nodes in a trusted manner. The sensed data collected at the IoT nodes were sent to the local nodes for processing and decision-making in the local algorithm placement scenario.

Two fog/MEC nodes with higher computational capabilities were deployed in iFogSim to represent the access tier. Each fog node in the access tier together with the fog blockchain connected to two local edge nodes in the local tier. In the simulation scenario, the data processing algorithm was placed in the access tier edge nodes and provided the necessary computational resources for each user’s data.

The core network contained the cloud server, which was the highest in available resources and responsible for the overall system management. In the scenario where the algorithm was placed at the core layer, the cloud server was responsible for running it.

The communication delay between the system components is presented in Figure 3.

Table 1 presents an overview of the simulation parameters used during the performance evaluation of the proposed framework. The parameters were defined according to a literature review of typical devices and networks used in similar application scenarios [50,51,55]. In this paper, we used the consortium blockchain for the proposed Health-BlockEdge framework. However, the iFogSim simulator (used in this paper) does not support the feature of modeling and simulating any particular type of blockchain. Therefore, to analyze the overall network performance of the proposed system, we used the parameters describing blockchain preprocessing power allocation and the number of instructions (MI), handled by each blockchain module, from [55]. The simulation parameters are illustrated in Table 1, where blockchain devices with different capacities and capabilities were considered in the simulation.

In the following, we present the simulation results for the key performance parameters.

### 6.2. Latency

**Figure 4:**
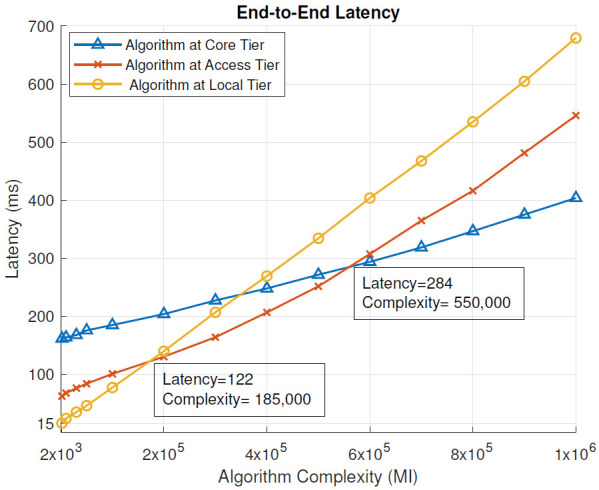
End-to-end latency: without the blockchain.

Without the blockchain: Figure 4 presents the end-to-end latency in milliseconds (ms) for different complexities of the analysis algorithm (millions of instructions, MI), in scenarios where the algorithm was placed at different tiers of the cloud IoT architecture and when blockchain pre-processing was not used. End-to-end latency is defined by (1). From the results, we can see that for low-complexity tasks (below 185,000 MI), the local tier, i.e., a local computing node, provided the lowest end-to-end latency and therefore the most optimal placement for the analysis algorithm. This is the region on the left side of the junction of yellow and red lines of Figure 4. When the algorithm complexity was moderate, between 185,000 MI and 550,000 MI, the most optimal tier for its placement was the access tier, i.e., the MEC server. In Figure 4, this is the region between the red/yellow line and red/blue line junctions. For high algorithm complexity, above 550,000 MI, the core tier, i.e., data center, provided the lowest end-to-end latency of the task execution. In Figure 4, this is the region on the right side of the red/blue line junction.

**Figure 5:**
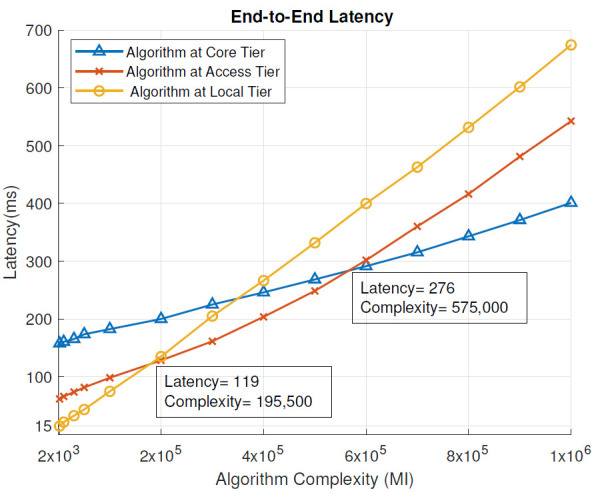
End-to-end latency: with the blockchain.

With Blockchain: Similarly to Figure 4, Figure 5 shows the end-to-end latency (ms) for different complexities of the analysis algorithm (MI), in scenarios where the algorithm was placed at different tiers of the cloud IoT architecture, when blockchain pre-processing was used. In this case, the local tier provided the lowest end-to-end latency, when the analysis algorithm complexity was below 195,500 MI. With the algorithm complexity between 195,500 and 575,000 MI, the MEC server (access tier) had the optimal placement for the algorithm. With the algorithm complexity above 575,000 MI, the data center (core tier) became the most optimal placement for the algorithm.

When blockchain pre-processing was used, the local tier remained the most suitable for slightly more complex algorithms (195,500 MI vs. 185,000 MI) compared to the scenario without blockchain-pre-processing. Overall, the end-to-end latency decreased slightly as the blockchain was introduced for per-processing at each tier of the network design.

### 6.3. Power Consumption

**Figure 6:**
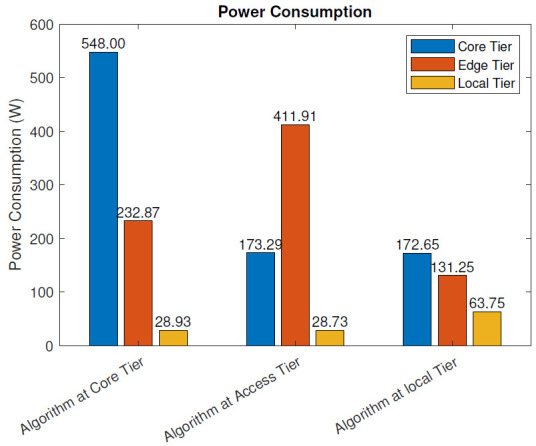
Power consumption: without the blockchain.

Without the blockchain: The power consumption (2) of the three different IoT algorithm placement scenarios, where blockchain pre-processing was not used, is presented in Figure 6. In the figure, we present the power consumption of each of the tree network tiers for each deployment scenario. When the analysis algorithm was placed at the data center, the core tier power consumption was 810.27 W, the access tier consumption 232.87 W, and local tier consumption 28.93 W. In this scenario, the power consumption consisted of the algorithm processing and communication costs at the core tier and only the communication cost at the lower tiers. The calculations excluded the power consumption of the sensing and actuation functionalities of the IoT nodes at the local tier, since they remained intact despite the location of the algorithm. The total power consumption of this deployment scenario was 810.27 W.

When the algorithm was run on an MEC server at the access tier, the power consumption was distributed as follows. The core tier power consumption was 173.29 W, by keeping the reserved data center resource idle. This tier took care of the service management and, therefore, needed to be active, even when the analysis algorithm was deployed on the lower tiers. In this scenario, the access tier resources (MEC server) ran the algorithm, and therefore, the computational load was focused on this tier. Together with the communication cost, the power consumption on the access tier was 411.91 W. At the local tier, the power consumption was 28.73 W. At this tier, only the communication costs were present in this scenario (in addition to the consumption from IoT sensing and actuation functions, which were excluded from the calculation). The total power consumption of this deployment scenario was 613.93 W.

When the algorithm was run in a local node at the local tier, the power consumption distributed as follows: 172.65 W at the core tier, 131.25 W at the access tier, and 63.75 W at the local tier. In this scenario, the local tier included the computational cost at the local node running the algorithm and the communication costs between it and the IoT devices with sensing and actuation functionalities. The access and core tier computational and networking elements were idle. The total power consumption of this deployment scenario was 367.65 W.

When comparing the three deployment scenarios, it can be clearly seen that when the distance between the sensing and actuation nodes and the node running the analysis algorithm increased, the total power consumption increased. Since our use case was data-intensive, containing, e.g., a high-definition video feed from the sensing device to the analysis algorithm, the distance between the source of the data and the processing node significantly affected the total power consumption.

**Figure 7:**
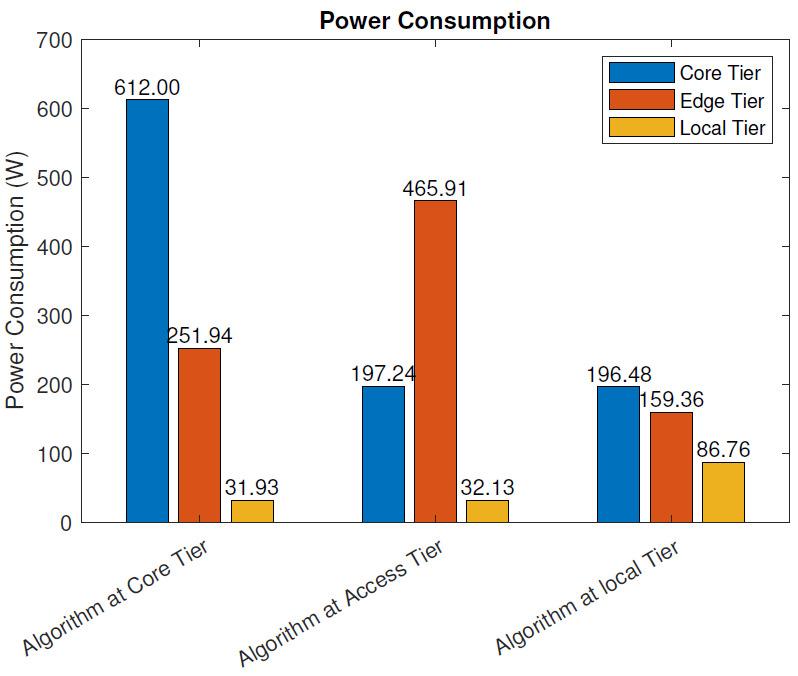
Power consumption: with the blockchain.

With the blockchain: Figure 7 depicts the power consumption of three IoT models when blockchain pre-processing was used. When the analysis algorithm was placed at the core tier (data center), the total power consumption was 895.87 W. The core tier power consumption was 612.00 W, the access tier consumption 251.94 W, and local tier consumption 31.93 W. When the analysis algorithm was placed at the access tier (MEC server), the total power consumption was 695.28 W, consisting of the core tier power consumption of 197.24 W, the access tier consumption of 465.91 W, and local tier consumption of 32.13 W. When the analysis algorithm was placed at the local tier (local node), the total power consumption was 442.60 W, consisting of the core tier power consumption 196.48 W, the access tier consumption 159.36 W, and the local tier consumption 86.76 W.

Similar to the scenario without blockchain pre-processing, the total power consumption was significantly affected by the distance between the sensing and actuation nodes and the node running the analysis algorithm.

The blockchain pre-processing increased the power consumption in all deployment scenarios: 10.6% when the algorithm was deployed at the core tier, 13.3% when the algorithm was deployed at the access tier, and 20.4% when the algorithm was deployed at the local tier.

### 6.4. Network Usage

**Figure 8:**
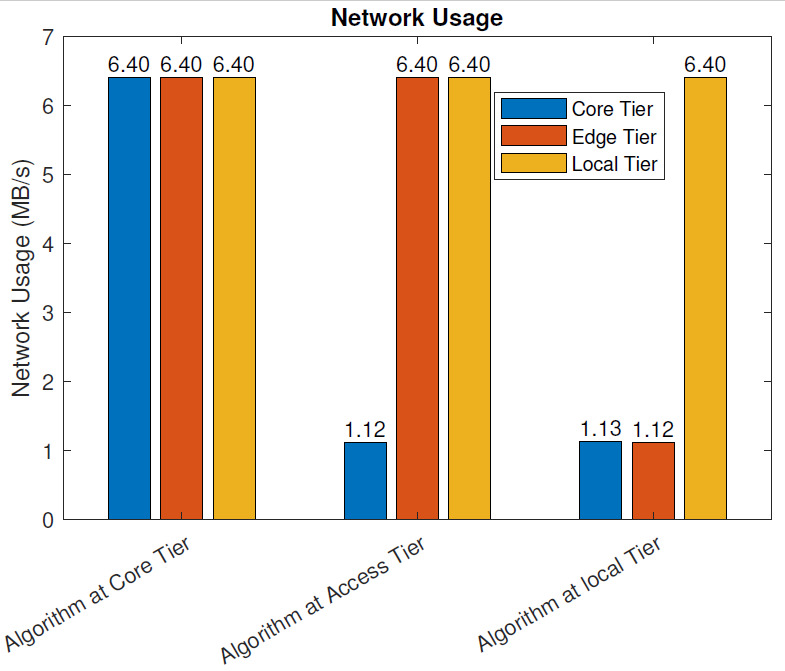
Network usage: without the blockchain.

Without the Blockchain: Figure 8 illustrates the network usage of the three IoT models in MB/s when blockchain pre-processing was not in use. In our use case, the raw data from various sensors, including the full-HD video monitoring feed (constant bit-rate of 1080p video, H.264 compression, and 40 FPS), as well as the necessary control data were exchanged between the sensor and actuator nodes and the computational node hosting the algorithm analyzing the sensor data. In addition, the main service at the data center communicated with the computational node hosting the algorithm analyzing the sensor data. These data included the exchange of the analyzed and processed data and control data. When the data were analyzed at the core tier (data center), the network utilization in each of the three tiers was 6.4 MB/s (all data, including control and raw data, were delivered through the whole data path between the IoT nodes and the data center). When the data were analyzed at the access tier (raw data delivered from IoT nodes to the MEC server), the network utilization at the local and access tiers was 6.4 MB/s, and the network utilization between the access and core tiers was 1.12MB/s. When the data were analyzed at the local tier (raw data not moved outside the local network), the network utilization at the local tier was 6.4 MB/s, 1.12 MB/s at the access tier, and 1.13 MB/s at the core tier.

**Figure 9:**
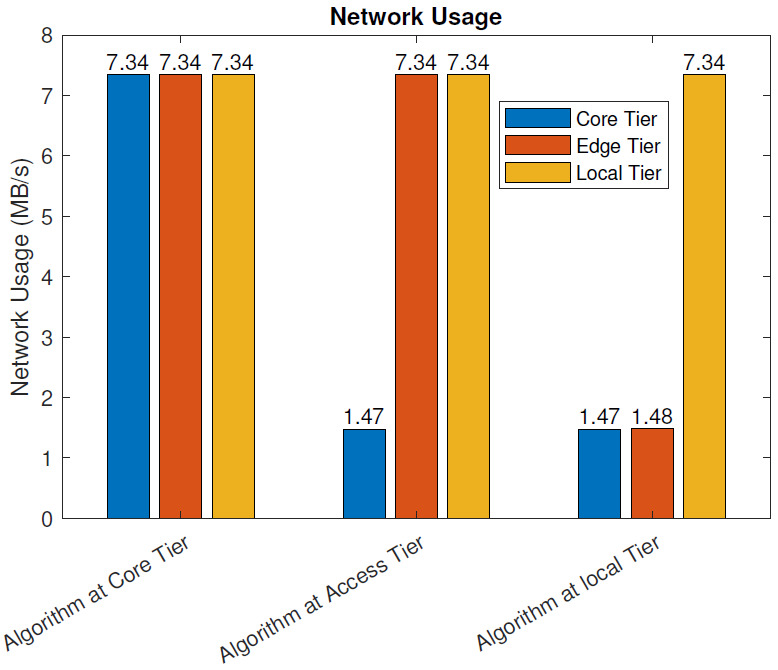
Network usage: with the blockchain.

With the blockchain: Figure 9 depicts the network usage of the three IoT models in MB/s when blockchain pre-processing was in use. As with the previous case, the raw data from various sensors, including the full-HD video monitoring feed, as well as the necessary control data, were exchanged between the sensor and actuator nodes and the computational node hosting the algorithm analyzing the sensor data. In addition, the main service at the data center communicated with the computational node hosting the algorithm analyzing the sensor data, including the exchange of the analyzed and processed data and control data. Furthermore, the parts of the distributed blockchain architecture, operating at all tiers of the architecture, exchanged data between each other and the operational entities.

When the data were analyzed at the core tier (data center), the network utilization in each of the three tiers was 7.34 MB/s. In this scenario, all data, including control and raw data, were delivered through the whole data path between the IoT nodes and the data center. In addition, the extra network utilization from the blockchain operation was included. When the data was analyzed at the access tier, the network utilization at the local and access tiers was 7.34 MB/s, and the network utilization between the access and core tiers was 1.47MB/s. When the data were analyzed at the local tier, the network utilization at the local tier was 7.34 MB/s, 1.48 MB/s at the access tier, and 1.47 MB/s at the core tier.

### 6.5. Total Cost

**Figure 10:**
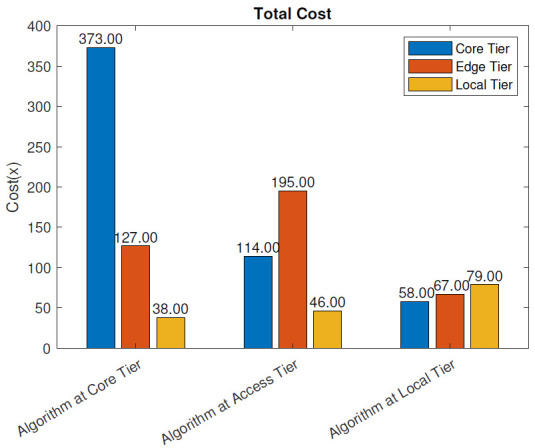
Total cost: without the blockchain.

Without the blockchain: The total operational cost, as defined in (3)–(5), of the different deployment scenarios without blockchain pre-processing is presented in Figure 10. The total cost was highest when the analysis algorithm was placed at the core tier, and it amounted to 538, compared to 355 when the algorithm was deployed at the access tier. The least costly solution was placing the algorithm at the local tier. In this case, the total cost was 204. In each scenario, the largest portion of the cost came from the tier where the algorithm was placed, as expected. When the application was placed at the access tier, the total cost decreased by 34.0% compared to the cloud placement. When the algorithm was moved further down to the local tier, the total cost decreased by 62.1% compared to the cloud placement. Therefore, placing the analysis algorithm at the local tier significantly reduced the total cost of the system compared to the access (MEC server) and core (data center) tier solutions.

**Figure 11:**
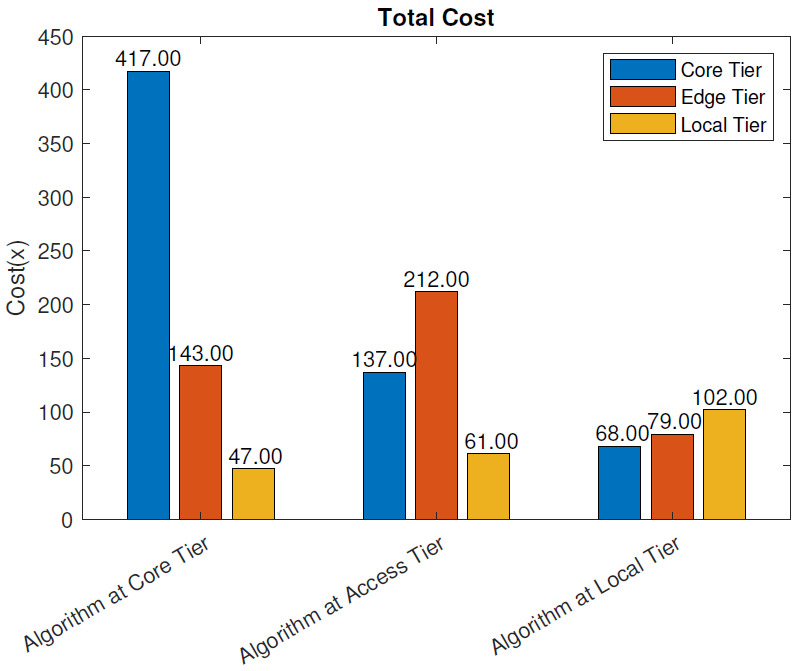
Total cost: with the blockchain.

With the blockchain: Figure 11 presents the total cost, as defined in (3), for the three algorithm placement scenarios when the blockchain pre-processing was used. Similar to the scenario without the blockchain, the total cost was largest when the analysis algorithm was placed at the core tier, 607, while the access tier placement scenario cost amounted to 410. The least costly solution was again placing the algorithm at the local tier where the total cost was 249. In each scenario, the largest portion of the cost came from the tier where the algorithm was placed. The reduction in cost when the algorithm was moved from the core tier to the access tier was 32.5% and when moved to the local tier, 59%. Having the blockchain pre-processing in the system slightly increased the total cost. At the core tier, the placement cost increased by 12.8%, at access tier by 15.5%, and at the local tier by 22%.

### 6.6. Number of Operations Executed

**Figure 12:**
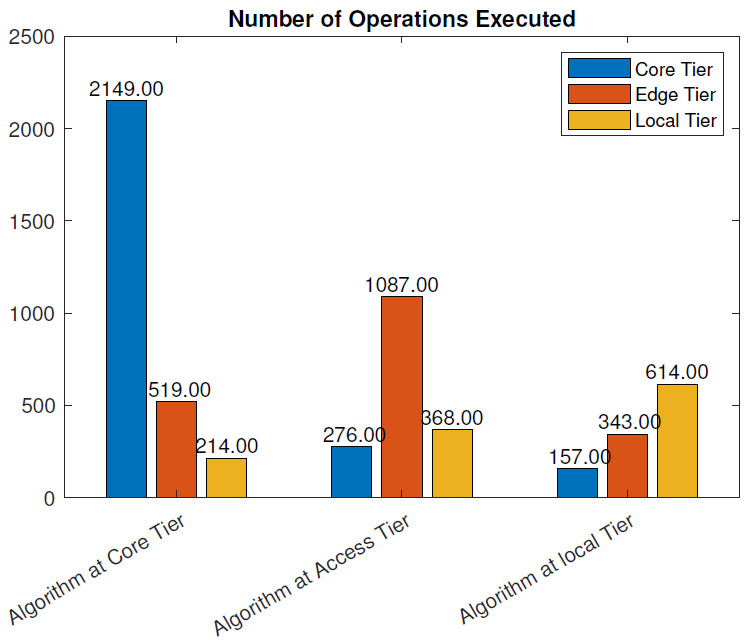
Number of operations executed: without the blockchain.

Without the blockchain: The number of operations executed at each layer of the three scenarios is given in Figure 12, in case no blockchain was used.

In the cloud placement scenario, the sum of all the operations necessary to perform N tasks was 2882. This included data processing and routing, resource management, control operations in the network, etc.

With the access tier placement, the number of operations executed was 1731 while at the local tier, it was 1114. Placing the algorithm further from the end-device significantly increased the number of instructions necessary for the execution of the same tasks, due to the communication overhead and the larger number of devices involved in data and control message forwarding. In the access tier placement, the number of control operations executed at the core layer decreased from 2149 to 276 instructions in the cloud tier placement. This was because the core layer only performed communication operations between the cloud and edge server and forwarding operations. The number of operations executed at the access layer included algorithm execution, data forwarding, and network communication operations. When the algorithm was placed at the local tier, the number of operations executed at local layer was 614. This included data processing, resource management, and control operations. On the other hand, the number of operations at the core and access tiers was much smaller and accounted for system control and orchestration.

**Figure 13:**
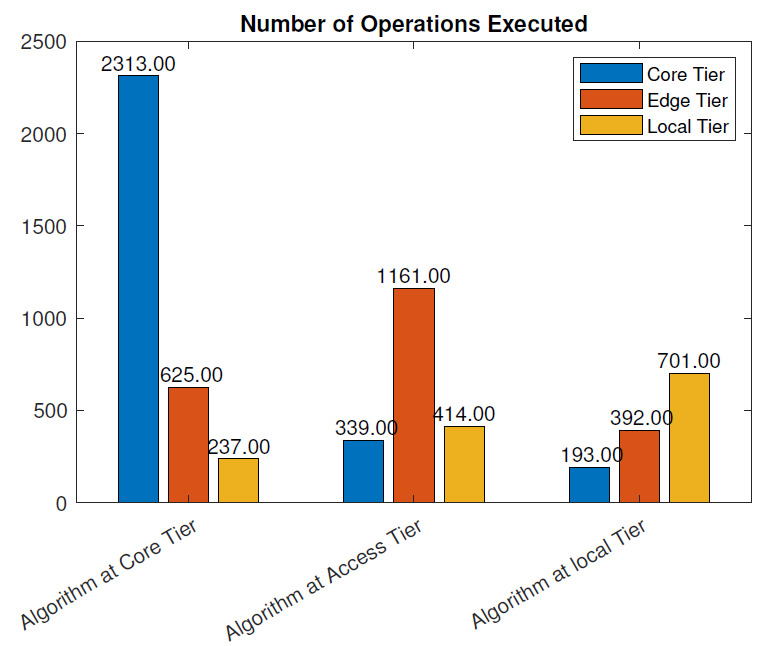
Number of operations executed: with the blockchain.

With the blockchain: Figure 13 illustrates the number of operations executed in the three placement scenarios when the blockchain was used. If the algorithm was placed at the core tier, the total number of operations executed within the local network was 3175. Compared to the scenario without blockchain, there was a 10.1% increase in the number of instructions in this case. With the access tier placement, the total number of instructions was 1914, which was a 10.5% increase compared to the scenario without the blockchain. Finally, the total number of instruction executed when the algorithm was run at the local tier was 1286, which was a 15.4% increase compared to the implementation without the blockchain.

### 6.7. Summary of the Results

In this section, we evaluate the impact of using the blockchain in a remote healthcare monitoring use case on the performance, efficiency, and resource utilization in three IoT edge cloud deployment scenarios. The main observations were as follows:

- From the latency evaluation (Figure 4 and Figure 5), we can see that the use of blockchain pre-processing did not have a significant effect on the latencies. The optimal regions for each deployment scenario were changed by 5–6% towards favoring more local setups.
- From the power consumption evaluation (Figure 6 and Figure 7), we can see that blockchain pre-processing increased the power consumption by 10.6% when the algorithm was deployed at the core tier, 13.3% when deployed at the access tier, and 20.4% when deployed at the local tier.
- When comparing the network usage (Figure 8 and Figure 9), we can see that the overall network usage was increased by roughly 15–31% when blockchain pre-processing was used.
- With respect to the total operational cost (the sum of communication and computation cost; Figure 10 and Figure 11), the blockchain pre-processing increased the total cost by 12.8% when processing was done at the core tier, by 15.5% when the processing was done at the access tier by, and by 22% when the processing was done locally.
- When comparing the number of computational operations executed at different levels of the architecture (Figure 12 and Figure 13), blockchain pre-processing increased the total number of instructions by 10.1%, when the processing was done at the core tier, by 10.5% when the processing was done at the access tier by, and by 15.4% when the processing was done locally.

## 7. Discussion and Future Directions

In this paper, we dealt with two highly important enabling technologies for digital healthcare: edge computing and the blockchain. The integration of the blockchain with edge computing is considered vital to providing secure, trusted, and delay-critical healthcare services for remote monitoring scenarios, e.g., elderly home care. The proposed conceptual Health-BlockEdge framework brings computational and processing resources near to the end-users and devices to perform necessary real-time data analysis and decision-making functions and to improve system-level resource efficiency.

The consortium blockchain technology used in this paper improves the overall security of remote health monitoring systems by providing key features such as confidentiality, transparency, immutability, traceability, data privacy, and availability [41,56].The inclusion of the blockchain also enables various highly important characteristics for the selected healthcare use case, e.g., trusted data sharing, secure monitoring or tracking of different processes and their phases, and keeping the electronic medical records of users.

The proposed blockchain-edge approach (Health-BlockEdge concept) improves the data privacy protection by limiting the propagation of sensitive data at the local and edge networks instead of sending all data to the cloud. Our proposed framework does this in two ways. First, since we can provide analysis capability at the edge, not all private data need to pass through the public (cloud) servers. Second, data that need to be processed on public servers can be anonymized at the edge. In addition, the blockchain can also fulfill the anonymity requirements of end-users by preventing the leakage of their real identities to the access/edge and core tiers. For example, similar anonymous settings can be adopted as the authors developed in [57], where they proposed blockchain-based anonymous authentication mechanisms for edge computing-based smart grid systems.

We evaluated the performance and efficiency of our proposed framework in the three IoT edge cloud deployment scenarios and compared the results with the case where the blockchain was not in use. According to our results, the end-to-end latency decreased slightly as the blockchain was introduced for pre-processing at each tier of the network design. With respect to power consumption, the blockchain pre-processing increased the power consumption by roughly 10–20%, depending on which tier of operation the data analysis functions were deployed. The blockchain pre-processing increased the system overhead with respect to the combined effect of computational and communication cost by roughly 13–22% and the number of computational instructions on the system level by roughly 10–15%. In conclusion, when considering the potential of blockchain usage in improving the security, privacy, and trust in healthcare monitoring scenarios, the increase in the system cost can be considered tolerable.

While evaluating the performance of the proposed system, we also observed some potential limitations of this work. For example, in our setup of the simulations, we were not able to model the actual blockchain (consortium) blocks because the iFogSim simulator does not provide any features for the blockchain. Therefore, we only considered the blockchain processing power and the number of blockchain instructions (MI) handled by each blockchain module for the performance evaluation of our proposed framework. By simulating or implementing the actual blockchain model in this three-tiered architecture, the analysis of the overall network performance could be improved.

The proposed framework also represents the infrastructural bases for enabling future healthcare services such as remote health monitoring and contactless patient treatment. These innovations aim at reducing the price of healthcare services and improving the availability of healthcare. The proposed framework in this paper can be extended to interesting avenues for future work, e.g., artificial intelligence (AI)-based optimization of the combined use of the blockchain and edge computing in the healthcare domain for improved performance and efficiency. Another direction for future work is to develop solutions to maximally utilize the features of the blockchain in bringing trust between different stakeholders of complex distributed healthcare communication and data management systems.

## 8. Conclusions

The rapid evolution of technology allows the healthcare sector to adopt intelligent, context-aware, secure, and ubiquitous healthcare services. It has become highly important to propose value-creating, yet cost-efficient digital solutions to healthcare systems. These solutions should provide effective means of healthcare services in both hospital and home care scenarios. The blockchain is a promising technology for enabling a trustful distributed computing environment in the context of future healthcare. However, despite its many desirable features, there are still concerns related to, e.g., the performance and efficiency of blockchain technologies.

In this paper, we addressed these challenges by integrating blockchain with edge computing to cope with some of the key requirements of smart remote healthcare systems, such as long operating times, low cost, resilience to network problems, security, and trust in highly dynamic network conditions. Through simulations of our proposed Health-BlockEdge concept, we evaluated the performance of our approach in terms of latency, power consumption, network utilization, and computational load, compared to a scenario where no blockchain was used.

According to the results, our concept demonstrated the feasibility of the combined use of blockchain and edge computing to provide decentralized trust, reliable real-time access, and control of the network and computational capacity in the digital healthcare environment, without compromising the system performance and resource efficiency.

## Figures and Tables

**Table 1:** Simulation parameters for the Health-BlockEdge framework. MI, millions of instructions.

Parameters	Data Center	MEC Server	Edge Nodes	IoT Nodes
	(Core Tier)	(Access Tier)	(Local Tier)	(Local Tier)
Upstream bandwidth (MBps)	140	70	34	10.5
Downstream bandwidth (MBps)	70	35	16	8
Storage capabilities/RAM (GB)	16	8	4	1
Processing capabilities/CPU (MIPS)	15,000–20,000	8000–13,500	3500–7500	400–1300
Communication latency (ms)	150	50	8	1
Blockchain instructions (MI)	25	14	8	-
Blockchain processing power (Idle-Max) (W)	20–80	12–40	1.4–20	-

## Data Availability

Not Applicable.

## References

[1] Jayaraman P.P., Forkan A.R.M., Morshed A., Haghighi P.D., Kang Y.B. (2020). Healthcare 4.0: A review of frontiers in digital health. Wiley Interdiscip. Rev. Data Min. Knowl. Discov..

[2] Ahmad I., Shahabuddin S., Kumar T., Okwuibe J., Gurtov A., Ylianttila M. (2019). Security for 5G and Beyond. IEEE Commun. Surv. Tutor..

[3] Kumar T., Liyanage M., Ahmad I., Braeken A., Ylianttila M. (2018). User Privacy, Identity and Trust in 5G. A Comprehensive Guide to 5G Security.

[4] Mohanta B., Das P., Patnaik S. Healthcare 5.0: A Paradigm Shift in Digital Healthcare System Using Artificial Intelligence, IoT and 5G Communication. Proceedings of the 2019 International Conference on Applied Machine Learning (ICAML).

[5] Alam M.M., Malik H., Khan M.I., Pardy T., Kuusik A., Le Moullec Y. (2018). A Survey on the Roles of Communication Technologies in IoT-Based Personalized Healthcare Applications. IEEE Access.

[6] Javaid M., Khan I.H. (2021). Internet of Things (IoT) enabled healthcare helps to take the challenges of COVID-19 Pandemic. J. Oral Biol. Craniofacial Res..

[7] Ijaz M.F., Attique M., Son Y. (2020). Data-driven cervical cancer prediction model with outlier detection and over-sampling methods. Sensors.

[8] Qadri Y.A., Nauman A., Zikria Y.B., Vasilakos A.V., Kim S.W. (2020). The Future of Healthcare Internet of Things: A Survey of Emerging Technologies. IEEE Commun. Surv. Tutor..

[9] Nikou S., Agahari W., Keijzer-Broers W., de Reuver M. (2020). Digital healthcare technology adoption by elderly people: A capability approach model. Telemat. Inform..

[10] Aloi G., Fortino G., Gravina R., Pace P., Savaglio C. (2020). Simulation-driven platform for Edge-based AAL systems. IEEE J. Sel. Areas Commun..

[11] Bajenaru L., Ianculescu M., Dobre C. A Holistic Approach for Creating a Digital Ecosystem Enabling Personalized Assistive Care for Elderly. Proceedings of the 2018 IEEE 16th International Conference on Embedded and Ubiquitous Computing (EUC).

[12] Hossain M.S., Muhammad G., Guizani N. (2020). Explainable AI and Mass Surveillance System-Based Healthcare Framework to Combat COVID-I9 Like Pandemics. IEEE Netw..

[13] Li X., Huang X., Li C., Yu R., Shu L. (2019). EdgeCare: Leveraging Edge Computing for Collaborative Data Management in Mobile Healthcare Systems. IEEE Access.

[14] Ali F., El-Sappagh S., Islam S.R., Kwak D., Ali A., Imran M., Kwak K.S. (2020). A smart healthcare monitoring system for heart disease prediction based on ensemble deep learning and feature fusion. Inf. Fusion.

[15] Shi W., Cao J., Zhang Q., Li Y., Xu L. (2016). Edge Computing: Vision and Challenges. IEEE Internet Things J..

[16] Malik S.U., Akram H., Gill S.S., Pervaiz H., Malik H. (2020). EFFORT: Energy efficient framework for offload communication in mobile cloud computing. Softw. Pract. Exp..

[17] McGhin T., Choo K.K.R., Liu C.Z., He D. (2019). Blockchain in healthcare applications: Research challenges and opportunities. J. Netw. Comput. Appl..

[18] Ramani V., Kumar T., Bracken A., Liyanage M., Ylianttila M. Secure and Efficient Data Accessibility in Blockchain Based Healthcare Systems. Proceedings of the 2018 IEEE Global Communications Conference (GLOBECOM).

[19] Kumar T., Ramani V., Ahmad I., Braeken A., Harjula E., Ylianttila M. Blockchain Utilization in Healthcare: Key Requirements and Challenges. Proceedings of the 2018 IEEE 20th International Conference on e-Health Networking, Applications and Services (Healthcom).

[20] Kassab M.H., DeFranco J., Malas T., Laplante P., Destefanis G., Graciano Neto V.V. (2019). Exploring Research in Blockchain for Healthcare and a Roadmap for the Future. IEEE Trans. Emerg. Top. Comput..

[21] Yousefpour A., Fung C., Nguyen T., Kadiyala K., Jalali F., Niakanlahiji A., Kong J., Jue J.P. (2019). All One Needs to Know about Fog Computing and Related Edge Computing Paradigms. J. Syst. Archit..

[22] Hartmann M., Hashmi U.S., Imran A. (2019). Edge computing in smart health care systems: Review, challenges, and research directions. Trans. Emerg. Telecommun. Technol..

[23] Abbas N., Zhang Y., Taherkordi A., Skeie T. (2017). Mobile edge computing: A survey. IEEE Internet Things J..

[24] Bonomi F., Milito R., Zhu J., Addepalli S. Fog Computing and Its Role in the Internet of Things. Proceedings of the First Edition of the MCC Workshop on Mobile Cloud Computing.

[25] Harjula E., Karhula P., Islam J., Leppänen T., Manzoor A., Liyanage M., Chauhan J., Kumar T., Ahmad I., Ylianttila M. (2019). Decentralized IoT Edge Nanoservice Architecture for Future Gadget-Free Computing. IEEE Access.

[26] Khan O.A., Malik S.U., Baig F.M., Islam S.U., Pervaiz H., Malik H., Ahmed S.H. (2020). A cache-based approach toward improved scheduling in fog computing. Softw. Pract. Exp..

[27] Davies A. (2014). Cisco Pushes IoT Analytics to the Extreme Edge with Mist Computing. https://rethinkresearch.biz/articles/cisco-pushes-iot-analytics-extreme-edge-mist-computing-2/.

[28] Preden J.S., Tammemäe K., Jantsch A., Leier M., Riid A., Calis E. (2015). The benefits of self-awareness and attention in fog and mist computing. Computer.

[29] Barik D.R., Dubey A., Tripathi A., Pratik T., Sasane S., Lenka R., Dubey H., Mankodiya K., Kumar V. (2018). Mist Data: Leveraging Mist Computing for Secure and Scalable Architecture for Smart and Connected Health. Procedia Comput. Sci..

[30] Bhattacharya S., Senapati S., Soy S.K., Misra C., Barik R.K. Performance Analysis Of Enhanced Mist-Assisted Cloud Computing Model For Healthcare System. Proceedings of the 2020 International Conference on Computer Science, Engineering and Applications (ICCSEA).

[31] Monrat A.A., Schelén O., Andersson K. (2019). A Survey of Blockchain From the Perspectives of Applications, Challenges, and Opportunities. IEEE Access.

[32] Nakamoto S. (2019). Bitcoin: A Peer-to-Peer Electronic Cash System; Technical Report; Manubot. https://www.markdownguide.org/.

[33] Xie J., Tang H., Huang T., Yu F.R., Xie R., Liu J., Liu Y. (2019). A Survey of Blockchain Technology Applied to Smart Cities: Research Issues and Challenges. IEEE Commun. Surv. Tutor..

[34] Wu M., Wang K., Cai X., Guo S., Guo M., Rong C. (2019). A Comprehensive Survey of Blockchain: From Theory to IoT Applications and Beyond. IEEE Internet Things J..

[35] Gamage H., Weerasinghe H., Dias N. (2020). A survey on blockchain technology concepts, applications, and issues. SN Comput. Sci..

[36] De Aguiar E.J., Faiçal B.S., Krishnamachari B., Ueyama J. (2020). A survey of blockchain-based strategies for healthcare. ACM Comput. Surv. (CSUR).

[37] Fekih R.B., Lahami M. Application of Blockchain Technology in Healthcare: A Comprehensive Study. Proceedings of the International Conference on Smart Homes and Health Telematics.

[38] Agbo C.C., Mahmoud Q.H. (2019). Comparison of blockchain frameworks for healthcare applications. Internet Technol. Lett..

[39] Khezr S., Moniruzzaman M., Yassine A., Benlamri R. (2019). Blockchain technology in healthcare: A comprehensive review and directions for future research. Appl. Sci..

[40] Katuwal G.J., Pandey S., Hennessey M., Lamichhane B. (2018). Applications of blockchain in healthcare: Current landscape & challenges. arXiv.

[41] Yaqoob I., Salah K., Jayaraman R., Al-Hammadi Y. (2020). Blockchain for healthcare data management: Opportunities, challenges, and future recommendations. Neural Comput. Appl..

[42] Abou-Nassar E.M., Iliyasu A.M., El-Kafrawy P.M., Song O., Bashir A.K., El-Latif A.A.A. (2020). DITrust Chain: Towards Blockchain-Based Trust Models for Sustainable Healthcare IoT Systems. IEEE Access.

[43] Shen B., Guo J., Yang Y. (2019). MedChain: Efficient healthcare data sharing via blockchain. Appl. Sci..

[44] Mkpa A., Chin J., Winckles A. Holistic Blockchain Approach to Foster Trust, Privacy and Security in IoT based Ambient Assisted Living Environment. Proceedings of the 2019 15th International Conference on Intelligent Environments (IE).

[45] Uddin M.A., Stranieri A., Gondal I., Balasubramanian V. (2021). A Survey on the Adoption of Blockchain in IoT: Challenges and Solutions. Blockchain Res. Appl..

[46] Mazlan A.A., Mohd Daud S., Mohd Sam S., Abas H., Abdul Rasid S.Z., Yusof M.F. (2020). Scalability Challenges in Healthcare Blockchain System—A Systematic Review. IEEE Access.

[47] Ejaz M., Kumar T., Ylianttila M., Harjula E. Performance and Efficiency Optimization of Multi-layer IoT Edge Architecture. Proceedings of the 2020 2nd 6G Wireless Summit (6G SUMMIT).

[48] Kumar T., Porambage P., Ahmad I., Liyanage M., Harjula E., Ylianttila M. (2018). Securing Gadget-Free Digital Services. Computer.

[49] Islam J., Harjula E., Kumar T., Karhula P., Ylianttila M. Enabled Virtualized Nanoservices for Local IoT Edge Networks. Proceedings of the 2019 IEEE Conference on Standards for Communications and Networking (CSCN).

[50] Bala M.I., Chishti M.A. Offloading in Cloud and Fog Hybrid Infrastructure Using iFogSim. Proceedings of the 2020 10th International Conference on Cloud Computing, Data Science & Engineering (Confluence).

[51] Sarkar I., Kumar S. Fog Computing Based Intelligent Security Surveillance Using PTZ Controller Camera. Proceedings of the 2019 10th International Conference on Computing, Communication and Networking Technologies (ICCCNT).

[52] Bala M.I., Chishti M.A. Optimizing the Computational Offloading Decision in Cloud-Fog Environment. Proceedings of the 2020 International Conference on Innovative Trends in Information Technology (ICITIIT).

[53] Kumar T., Harjula E., Ejaz M., Manzoor A., Porambage P., Ahmad I., Liyanage M., Braeken A., Ylianttila M. (2020). BlockEdge: Blockchain-Edge Framework for Industrial IoT Networks. IEEE Access.

[54] Kumar T., Braeken A., Ramani V., Ahmad I., Harjula E., Ylianttila M. SEC-BlockEdge: Security Threats in Blockchain-Edge Based Industrial IoT Networks. Proceedings of the 2019 11th International Workshop on Resilient Networks Design and Modeling (RNDM).

[55] Uddin M.A., Stranieri A., Gondal I., Balasurbramanian V. (2019). A Lightweight Blockchain Based Framework for Underwater IoT. Electronics.

[56] Tariq N., Qamar A., Asim M., Khan F.A. (2020). Blockchain and Smart Healthcare Security: A Survey. Procedia Comput. Sci..

[57] Wang J., Wu L., Choo K.R., He D. (2020). Blockchain-Based Anonymous Authentication With Key Management for Smart Grid Edge Computing Infrastructure. IEEE Trans. Ind. Inform..

